# SARS-CoV-2 within-host diversity and transmission

**DOI:** 10.1126/science.abg0821

**Published:** 2021-03-09

**Authors:** Katrina A. Lythgoe, Matthew Hall, Luca Ferretti, Mariateresa de Cesare, George MacIntyre-Cockett, Amy Trebes, Monique Andersson, Newton Otecko, Emma L. Wise, Nathan Moore, Jessica Lynch, Stephen Kidd, Nicholas Cortes, Matilde Mori, Rebecca Williams, Gabrielle Vernet, Anita Justice, Angie Green, Samuel M. Nicholls, M. Azim Ansari, Lucie Abeler-Drner, Catrin E. Moore, Timothy E. A. Peto, David W. Eyre, Robert Shaw, Peter Simmonds, David Buck, John A. Todd, on behalf of the Oxford Virus Sequencing Analysis Group (OVSG), Thomas R. Connor, Shirin Ashraf, Ana da Silva Filipe, James Shepherd, Emma C. Thomson, The COVID-19 Genomics UK (COG-UK) Consortium, David Bonsall, Christophe Fraser, Tanya Golubchik

**Affiliations:** 1 Big Data Institute, Nuffield Department of Medicine, University of Oxford, Old Road Campus, Oxford OX3 7LF, UK.; 2 Department of Zoology, University of Oxford, Oxford OX1 3SZ, UK.; 3 Wellcome Centre for Human Genetics, Nuffield Department of Medicine, NIHR Biomedical Research Centre, University of Oxford, Old Road Campus, Oxford OX3 7BN, UK.; 4 Oxford University Hospitals NHS Foundation Trust, John Radcliffe Hospital, Headington, Oxford OX3 9DU, UK.; 5 Division of Medical Virology, Stellenbosch University, Stellenbosch, South Africa.; 6 Hampshire Hospitals NHS Foundation Trust, Basingstoke and North Hampshire Hospital, Basingstoke RG24 9NA, UK.; 7 School of Biosciences and Medicine, University of Surrey, Guildford GU2 7XH, UK.; 8 Gibraltar Health Authority, Gibraltar, UK.; 9 School of Medicine, University of Southampton, Southampton SO17 1BJ, UK.; 10 Institute of Microbiology and Infection, University of Birmingham, Birmingham B15 2TT, UK.; 11 Peter Medawar Building for Pathogen Research, University of Oxford, Oxford OX1 3SY, UK.; 12 Nuffield Department of Medicine, University of Oxford, John Radcliffe Hospital, Headington, Oxford OX3 9DU, UK.; 13 Big Data Institute, Nuffield Department of Public Health, University of Oxford, Old Road Campus, Oxford OX3 7FL, UK.; 14 Pathogen Genomics Unit, Public Health Wales Microbiology, Cardiff CF10 4BZ, UK.; 15 Cardiff University School of Biosciences, Cardiff University, Cardiff CF10 3AX, UK.; 16 MRC-University of Glasgow Centre for Virus Research, Glasgow G61 1QH, UK.; 17 Wellcome Sanger Institute, Cambridge CB10 1SA, UK.

## Abstract

Extensive global sampling and sequencing of the pandemic virus severe acute respiratory syndrome coronavirus 2 (SARS-CoV-2) have enabled researchers to monitor its spread and to identify concerning new variants. Two important determinants of variant spread are how frequently they arise within individuals and how likely they are to be transmitted. To characterize within-host diversity and transmission, we deep-sequenced 1313 clinical samples from the United Kingdom. SARS-CoV-2 infections are characterized by low levels of within-host diversity when viral loads are high and by a narrow bottleneck at transmission. Most variants are either lost or occasionally fixed at the point of transmission, with minimal persistence of shared diversity, patterns that are readily observable on the phylogenetic tree. Our results suggest that transmission-enhancing and/or immune-escape SARS-CoV-2 variants are likely to arise infrequently but could spread rapidly if successfully transmitted.

## Patterns and bottlenecks

A year into the severe acute respiratory syndrome coronavirus 2 pandemic, we are experiencing waves of new variants emerging. Some of these variants have worrying functional implications, such as increased transmissibility or antibody treatment escape. Lythgoe *et al.* have undertaken in-depth sequencing of more than 1000 hospital patients' isolates to find out how the virus is mutating within individuals. Overall, there seem to be consistent and reproducible patterns of within-host virus diversity. The authors observed only one or two variants in most samples, but a few carried many variants. Although the evidence indicates strong purifying selection, including in the spike protein responsible for viral entry, the authors also saw evidence for transmission clusters associated with households and other possible superspreader events. After transmission, most variants fizzled out, but occasionally some initiated ongoing transmission and wider dissemination.

*Science*, this issue p. eabg0821

## Abstract (structured)

**INTRODUCTION:**

Genome sequencing at an unprecedented scale during the severe acute respiratory syndrome coronavirus 2 (SARS-CoV-2) pandemic is helping to track spread of the virus and to identify new variants. Most of this work considers a single consensus sequence for each infected person. Here, we looked beneath the consensus to analyze genetic variation within viral populations making up an infection and studied the fate of within-host mutations when an infection is transmitted to a new individual. Within**-**host diversity offers the means to help confirm direct transmission and identify new variants of concern.

**RATIONALE:**

We sequenced 1313 SARS-CoV-2 samples from the first wave of infection in the United Kingdom. We characterized within-host diversity and dynamics in the context of transmission and ongoing viral evolution.

**RESULTS:**

Within-host diversity can be described by the number of intrahost single nucleotide variants (iSNVs) occurring above a given minor allele frequency (MAF) threshold. We found that in lower-viral-load samples, stochastic sampling effects resulted in a higher variance in MAFs, leading to more iSNVs being detected at any threshold. Based on a subset of 27 pairs of high-viral-load replicate RNA samples (>50,000 uniquely mapped veSEQ reads, corresponding to a cycle threshold of ~22), iSNVs with a minimum 3% MAF were highly reproducible. Comparing samples from two time points from 41 individuals, taken on average 6 days apart (interquartile ratio 2 to 10), we observed a dynamic process of iSNV generation and loss. Comparing iSNVs among 14 household contact pairs, we estimated transmission bottleneck sizes of one to eight viruses. Consensus differences between individuals in the same household, where sample depth allowed iSNV detection, were explained by the presence of an iSNV at the same site in the paired individual, consistent with direct transmission leading to fixation. We next focused on a set of 563 high-confidence iSNV sites that were variant in at least one high-viral-load sample (>50,000 uniquely mapped); low-confidence iSNVs unlikely to represent genomic diversity were excluded. Within-host diversity was limited in high-viral-load samples (mean 1.4 iSNVs per sample). Two exceptions, each with >14 iSNVs, showed variant frequencies consistent with coinfection or contamination. Overall, we estimated that 1 to 2% of samples in our dataset were coinfected and/or contaminated. Additionally, one sample was coinfected with another coronavirus (OC43), with no detectable impact on diversity. The ratio of nonsynonymous to synonymous (*dN/dS*) iSNVs was consistent with within-host purifying selection when estimated across the whole genome [*dN/dS* = 0.55, 95% confidence interval (95% CI) = 0.49 to 0.61] and for the Spike gene (*dN/dS *= 0.60, 95% CI = 0.45 to 0.82). Nevertheless, we observed Spike variants in multiple samples that have been shown to increase viral infectivity (L5F) or resistance to antibodies (G446V and A879V). We observed a strong association between high-confidence iSNVs and a consensus change on the phylogeny (153 cases), consistent with fixation after transmission or de novo mutations reaching consensus. Shared variants that never reached consensus (261 cases) were not phylogenetically associated.

**CONCLUSION:**

Using robust methods to call within-host variants, we uncovered a consistent pattern of low within-host diversity, purifying selection, and narrow transmission bottlenecks. Within-host emergence of vaccine and therapeutic escape mutations is likely to be relatively rare, at least during early infection, when viral loads are high, but the observation of immune-escape variants in high-viral-load samples underlines the need for continued vigilance.

## Teaser

More than a thousand deep-sequenced clinical samples from the UK reveal that SARS-CoV-2 has limited genetic diversity within most individuals.

## Graphical abstract

**Figure:**
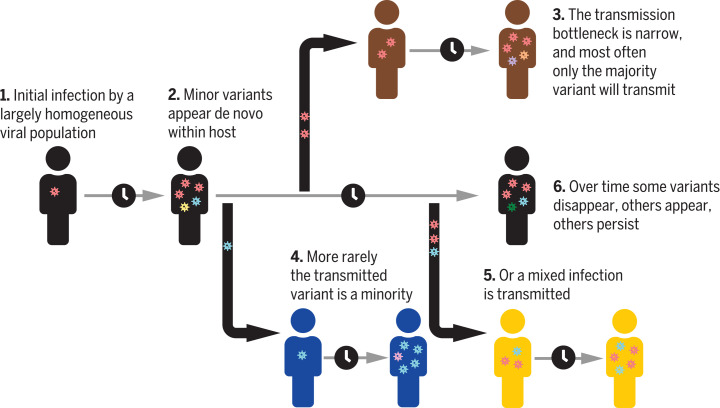
Diagram showing low SARS-CoV-2 within-host genetic diversity and narrow transmission bottleneck. Individuals with high viral load typically have few, if any, within-host variants. Narrow transmission bottlenecks mean that the major variant in the source individual was typically transmitted and the minor variants lost. Occasionally, the minor variant was transmitted, leading to a consensus change, or multiple variants were transmitted, resulting in a mixed infection. Credit: FontAwesome, licensed under CC BY 4.0.

The ongoing evolution of severe acute respiratory syndrome coronavirus 2 (SARS-CoV-2) has been the topic of considerable interest as the pandemic has unfolded. Clear lineage-defining single nucleotide polymorphisms (SNPs) have emerged (*1*), enabling tracking of viral spread (*2*, *3*) but also raising concerns that new mutations, or combinations of mutations, may confer selective advantages on the virus, hampering efforts at control. There is compelling evidence that the D614G mutation in the Spike protein (S), which spread globally during the first year of the pandemic, increases viral transmissibility (*4*,*6*). Current variants of concern include the B.1.1.7. lineage (*7*, *8*), with an estimated transmission advantage of ~50% (*9*), and the B.1.351 and P.1 lineages (*10*, *11*), which may have decreased sensitivity to natural and/or vaccine-acquired immunity (*12*,*14*). Lineage codes given here are as designated by Pangolin software (*1*).

Most analyses have been focused on mutations observed in viral consensus genomes, which represent the dominant variants within infected individuals. Ultimately though, new mutations emerge within individuals, so knowledge of the full underlying within-host diversity of the virus at the population level and how frequently this is transmitted is important for understanding adaptation and patterns of spread.

The United Kingdom experienced one of the most severe first waves of infection, with >1000 independent importation events contributing to substantial viral diversity during this period (*15*). In this study, we analyzed 1390 SARS-CoV-2 genomes from 1313 nasopharyngeal swabs sampled predominantly from symptomatic individuals on admission to the hospital and from health care workers during the first wave of infection (March to June 2020; table S1). The dataset comprised samples from 1173 unique individuals, including 41 with samples at two to four time points, plus 93 anonymous samples, with multiple RNA aliquots from 76/1313 samples resequenced to test for reproducibility. The samples were collected by two geographically separate hospital trusts located 60 km apart: Oxford University Hospitals and Basingstoke and North Hampshire Hospital. Using veSEQ, an RNA-Seq protocol based on a quantitative targeted enrichment strategy (*16*), which we previously validated for other viruses (*16*,*19*), we characterized the full spectrum of within-host diversity in SARS-CoV-2 and analyzed it in the context of the consensus phylogeny.

We observed low levels of intrahost diversity in high-viral-load samples, with evidence of within-host evolutionary constraint genome wide, including S. Although within-host variants could be observed in multiple individuals in the same phylogenetic cluster, some of whom resided in the same household, most viral variants were either lost, or occasionally fixed, at the point of transmission, with a narrow transmission bottleneck. These results suggest that during early infection, when viral loads are high and transmission is most likely (*20*,*22*), mutations that increase transmissibility or potential vaccine- or therapy-escape mutations may rarely emerge and subsequently transmit. Nonetheless, we identified variants present in multiple individuals that could affect receptor binding or neutralization by antibodies. Because the fitness advantage of escape mutations in populations that are highly vaccinated or have high levels of natural immunity could be substantial, and because mutational effects can depend on the genetic background on which they are found, these findings underline the need for continued vigilance and monitoring.

## Detection of variants is influenced by viral load

Reliable estimation of variant frequencies requires quantitative sequencing such that the number of reads is proportional to the amount of corresponding sequence in the sample of interest. The veSEQ protocol has been shown previously to be quantitative for a number of different pathogens (*17*), including respiratory viruses such as respiratory syncytial virus (RSV) (*18*). We demonstrated here that the same quantitative relationship holds for SARS-CoV-2. The number of uniquely mapped sequencing reads that we obtained rose log-log linearly with the number of RNA copies in serial dilutions of synthetic RNA controls (*r*^2 ^= 0.87; fig. S1A) and was consequently correlated with cycle threshold (Ct) values of clinical samples (fig. S1B), indicating that veSEQ reads can be considered a representative sample of viral sequences within the input RNA.

**Fig. 1:**
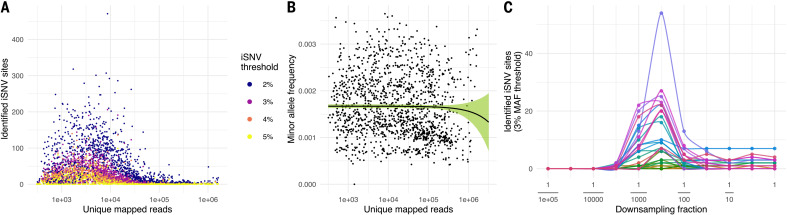
Characterization of iSNV frequencies. (**A**) Distribution of the number of identified iSNV sites in each sample against the number of unique mapped reads. The colors represent different MAF thresholds. An iSNV site is identified within a sample if the MAF is greater than the threshold. (**B**) Distribution of the mean MAF in each sample against the number of unique mapped reads, with no MAF threshold applied. The black line is the estimated mean value by linear regression. The green ribbon is the 95% CI. (**C**) Distribution of the number of identified iSNV sites at the 3% MAF threshold when subsampling from high-depth samples. Each color represents a different high-depth sample.

To understand within-host diversity, we quantified the number of intrahost single-nucleotide variants (iSNVs) in the full set of 1390 genomes, testing different thresholds for identifying variants of between 2 and 5% minor allele frequency (MAF). A minimum depth of at least 100 reads was also required to call an iSNV, and all sites with MAF greater than the threshold were included (Fig. 1A).

For all thresholds, we observed a nonlinear relationship between sample viral load (estimated by total unique mapped reads) and the number of detected iSNVs, with the highest number of iSNVs detected at intermediate viral loads (~2000 mapped reads). However, the mean MAF per sample did not vary with viral load when no threshold was applied (*P* = 0.291, linear regression; Fig. 1B). This indicates that as the number of mapped reads decreases, the variance in the observed MAF increases, whereas the mean stays the same. This effect is at least partially caused by the inverse relationship of the binomial distribution between the total number of draws and the variance in the proportion of successes observed among those draws. In Fig. 1C, we demonstrate this effect by down-sampling from high-depth samples: The increasing variance associated with sparser sampling causes the number of threshold-crossing iSNVs to increase until eventually so few reads are sampled that no iSNVs are detected.

This sampling effect of low viral load does not preclude the existence of biological mechanisms also contributing to greater intrahost diversity in low-viral-load samples. After the initial peak, viral loads typically decrease as infection progresses (*20*), whereas genetic diversity may increase, as observed in other viral infections such as HIV (*23*). RNA damage (*24*) as infection progresses could also contribute to the observed increased diversity in low-depth samples.

## Within-host variant frequencies are reproducible

To calibrate our variant calling and to minimize false discovery rates, we compared iSNVs in resequenced controls with data for the stock RNA sequenced and provided by the manufacturer (Twist Bioscience) and masked sites vulnerable to in vitro generation of variants (table S2). We also masked a further 18 sites that were observed to be variant (>3% MAF) in 20 or more high-viral-load samples (table S3 and fig. S3). Most had consistently low MAFs among samples, and some showed evidence of strand bias and/or low reproducibility between technical replicates (fig. S2), suggesting that they were not true genomic variants. Among the excluded sites was 11083, which was observed in 46 samples and is globally ubiquitous in GISAID (Global Initiative on Sharing All Influenza Data) data. From manual examination of mapped reads in our dataset, this appeared to be caused by a common miscalling of a within-host polymorphic deletion upstream at site 11082 occurring in a poly-T homopolymeric stretch. If genuine, then this homopolymer stutter may have a structural or regulatory role; however, methodological issues in resolving this difficult-to-map region cannot be ruled out.

**Fig. 2:**
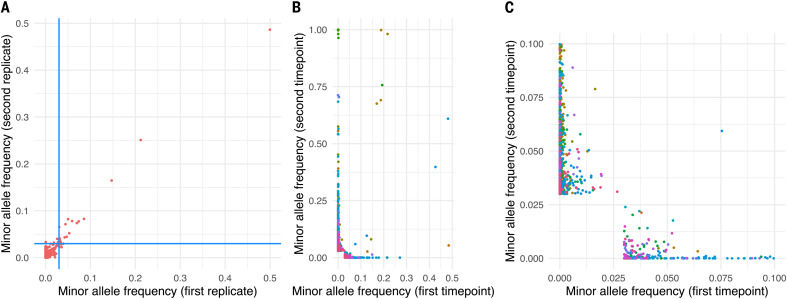
Comparison of allele frequencies between sequencing replicates of the same sample and multiple time points from the same individual. (**A**) Comparison of MAFs from 27 replicate pairs resequenced from RNA, with each point representing a single genomic position in a pair of replicates. The plot represents all MAF frequency comparisons for the 27 samples where both replicates had >50,000 unique mapped reads, limited to genomic sites with MAF >0.02 in at least one of the 54 replicates. The blue lines are the threshold value of 0.03. (**B** and **C**) Comparison of allele frequencies from 41 individuals sampled on different days, with each point representing a genomic position in a pair of samples from the same individual. Each individual is represented by a different color, and for each individual, all genomic positions are considered where the MAF >0.03 at either sampling time point and/or a change in consensus was observed. In all cases, the poly-A tail and sites variable in RNA synthetic controls were excluded, as were sites observed to be variable in >20 samples at MAF >3% because these are unlikely to represent genomic variants. (C) is an enlargement of the region of (B) near the origin.

Establishing reliable variant calling thresholds for clinical samples in which true variant frequencies are unknown ideally requires resequencing of multiple samples from RNA to test for concordance. Working within the constraints of small volumes of remnant RNA from laboratory testing, we resequenced 76 high-viral-load samples, of which 27 replicate pairs generated sufficient read numbers (>50,000 unique mapped reads) for reliable minor variant detection. iSNVs with <2% MAF were generally indistinguishable from noise, whereas those with 3% MAF were highly concordant between replicates (Fig. 2A and fig. S2).

## Within-host variants vary during infection

We also compared iSNV frequencies and consensus changes at different time points for the 41 multiply sampled individuals, with the duration between sampling ranging between 1 and 20 days apart (median 6 days; Fig. 2, B and C). Because viral loads tend to fall as infection progresses, we considered all samples rather than limiting ourselves to those with >50,000 unique mapped reads. Among the 41 individuals, we observed little concordance in minor variant frequencies across time points within individuals. Our observations, consistent with other studies (*24*,*26*), suggest a dynamic within-host landscape but also reflect the inherent stochasticity associated with low-viral-load samples.

## The transmission bottleneck size within households is small

The transmission bottleneck size is a key component in determining the likelihood that new within-host variants will spread in the population (*27*). Estimating bottleneck size is difficult for SARS-CoV-2 because it requires sufficient genetic diversity to differentiate distinct viruses that may be transmitted in known source-recipient pairs (*28*,*31*) and confidence that transmission is the cause of variants observed in both source and recipients. The inclusion of variants that are not shared by transmission can greatly increase transmission bottleneck size estimations (*29*). We identified 16 households in which two individuals had a first positive sample within 2 weeks of each other, and assumed direct transmission if the consensus sequences in the individuals had fewer than three differences (thus excluding one household). A further household was excluded because the assumed source individual had no variants with >3% MAF.

**Fig. 3:**
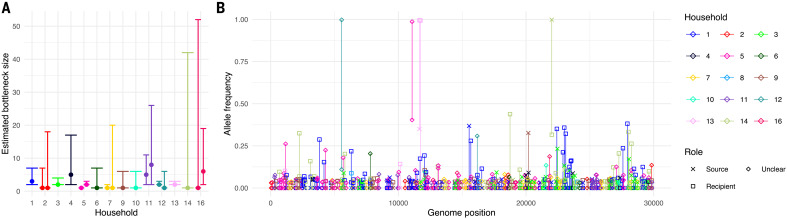
Small transmission bottleneck size within households. (**A**) Estimated bottleneck size in 14 households calculated using the exact beta-binomial method described in (*28*). Bottleneck size for both combinations of potential source and recipient were calculated if the first positive samples from each individual in the household were collected within a week of each other. No estimate was recorded if there were no identified iSNVs >3% MAF in the source individual (household 8) or if the two individuals in the household had more than two consensus differences (household 15). The error bars represent the 95% CI determined by the likelihood ratio test. (**B**) Fate of the identified iSNVs within households. Each line links the allele frequency of a given variant in one household member with that in the second member. Points and lines are colored by household. Each was identified as an iSNV in at least one individual but not necessarily (and usually not) both. Where the dates of sample collection differed by at least a week, we also indicate the assumed source and recipient members of the household.

Using the exact beta-binomial method (*28*), we estimated maximum likelihood bottleneck sizes between one and eight among the 14 household transmission pairs (Fig. 3A and table S4). These observations are consistent with the small bottleneck sizes observed for influenza (*30*,*32*) and SARS-CoV-2 (*33*,*37*) but considerably lower than estimates in a recent Austrian study (*25*). The reasons for the discrepancies are unclear but could reflect differences in how variants were selected for analysis (*37*) or how closely the observed diversity represents the diversity of virus both available for transmission and successfully transmitted. An association between the route of exposure and the transmission bottleneck has been demonstrated experimentally for influenza (*32*), so genuine differences in bottleneck sizes in different settings cannot be ruled out.

## Within-host variants are present in most SARS-CoV-2 samples

To further characterize iSNV sites within individuals, we identified a set of 563 high-confidence iSNV sites that were observed (i) in high-viral load samples with at least 50,000 unique mapped reads (462 samples, 160 from Oxford and 302 from Basingstoke), (ii) at a depth of at least 100 reads, (iii) with a MAF of at least 3%, and (iv) not observed to vary in synthetic RNA controls or to appear at low frequency in a large number of samples (table S3). All 1313 samples were included in our analysis under the assumption that by ascertaining on a small set of predefined sites, it is less likely that we included sites that only reach >3% MAF in low-viral-load samples because of the stochastic sampling effects described above.

**Fig. 4:**
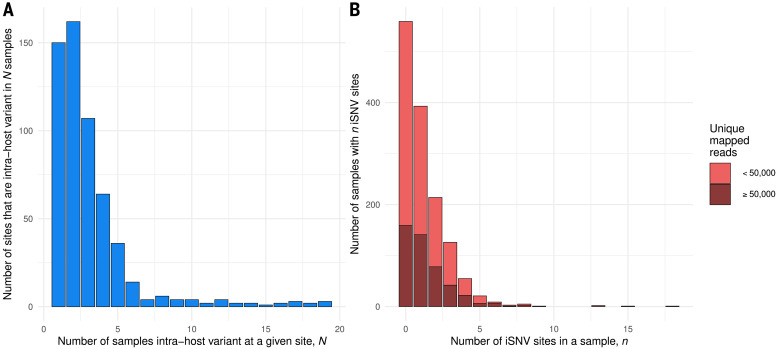
iSNV sites were often found in multiple samples and most samples had at least one iSNV. (**A**) Histogram showing the number iSNV sites that were found in *N* samples. All samples in our dataset are included. (**B**) Stacked histogram showing the number of samples that had *n* iSNV sites for all samples with >50,000 mapped reads (dark red) and samples with <50,000 mapped reads (light red). All 563 sites identified for variant analysis were included (see main text), including sites in the 3UTR and 5UTR but excluding the polyA tail and the 18 sites variable in 20+ individuals.

Among the iSNV sites taken forward for variant analysis, most were only observed in one or two of the 1313 samples (Fig. 4A), but most samples with >50,000 unique reads (305/462, 66%) harbored at least one iSNV (Fig. 4B). These low levels of SARS-CoV-2 within-host diversity during acute infection are consistent with other reported levels (*26*, *33*) but lower than in some other studies (*24*, *25*), likely reflecting how variants were identified.

Two samples had a particularly high number (15 and 18) of iSNVs, each with high and correlated MAFs consistent with coinfection by two diverse variant haplotypes (*38*). For one of these samples, laboratory contamination was unlikely because we could not identify any samples that could be the source. We could not distinguish between coinfection and contamination in the other sample because both variant haplotypes within it represented common genotypes in our study.

In general, however, the low level of genetic diversity of the virus makes identifying coinfection or contaminationand distinguishing between themdifficult. If sites where a large number of SNPs is present (mutations that distinguish common lineages in our dataset) are only observed to be variant within host because of coinfection or contamination, then we estimate that between ~1 and 2% of samples are potentially affected by coinfection or contamination (table S2). As a precaution against contamination or batch effects, we sequenced known epidemiologically linked samples in different batches where possible (fig. S4).

We hypothesized that a proportion of the observed within-host variation could have been due to coinfection with seasonal coronaviruses, which has been reported in 1 to 4% of SARS-CoV-2 infections (*39*, *40*). Specifically, closely matching reads from similar viruses could be mapped to SARS-CoV-2 and appear as mixed-base calls. To understand the impact of coinfection, we recaptured and analyzed a random subset of 180 samples spanning the full range of observed SARS-CoV-2 viral loads (Ct 14 to 33, median 19.8) using the Castanet multipathogen enrichment panel (*17*), which contains probes for all known human coronaviruses with the exception of SARS-CoV-2. Among the 111 samples that yielded both SARS-CoV-2 and Castanet data, we identified one sample that was also positive for another betacoronavirus, human coronavirus OC43 (fig. S5). Within the SARS-CoV-2 genome from this sample, which was complete and high-depth, we observed only a single iSNV at position 28580 and no evidence of mixed-base calls at any other genomic position. This suggests that even when coinfection was present, it did not affect the estimation of SARS-CoV-2 within-host diversity in our protocol. However, whether coinfection with OC43 or other coronaviruses exerts a selective pressure on SARS-CoV-2 remains an open question.

## Distribution of iSNVs across the genome

We next considered the distribution of the identified high-confidence iSNV sites across the genome. Even excluding the untranslated regions (UTRs), which have a highly elevated density of iSNV sites, there was considerable variability across the genome, with open-reading frames (ORFs) 3a, 7a, and 8 and nucleocapsid (N) showing the highest densities (Table 1). In addition, we calculated ratio of nonsynonymous to synonymous substitutions (*dN/dS*) values under the assumption that each iSNV appeared de novo in each individual in which it was observed (Table 1). Consistent with other studies (*24*, *33*), most areas of the genome appeared to be under purifying selection, with *dN/dS* values <1, including S. Without a full model incorporating within-host evolutionary dynamics and transmission, it is difficult to draw strong conclusions. However, we obtained similar results assuming that each iSNV was only generated once de novo and then subsequently transmitted (table S5). These patterns are also broadly consistent with *dN/dS* values calculated for SNPs among SARS-CoV-2 consensus genomes (*41*), suggesting that evolutionary forces at the within-host level are reflected at the between-host level, at least for within-host variant sites in high-viral-load samples.

**Table 1:** iSNVs and *dN/dS* by gene and over the whole genome.

**Gene**	**Length**	**iSNVs**			**Mean iSNVs** **per 100 sites**		***dN/dS*** **(95% CI)**
		**Total**	**NS**	**S**			
5UTR	265	82	-	-	0.0223		-
ORF1a	13218	572	369	203	0.0031		0.51 (0.43, 0.61)
nsp1	540	54	39	15	0.0072		0.79 (0.44, 1.47)
nsp2	1914	105	65	40	0.0039		0.46 (0.31, 0.69)
nsp3	5835	175	108	67	0.0022		0.45 (0.33, 0.61)
nsp4	1500	101	61	40	0.0048		0.44 (0.3, 0.66)
nsp5A	918	25	22	3	0.002		2.08 (0.72, 8.77)
nsp6	870	62	42	20	0.0051		0.58 (0.35, 1.01)
nsp7	249	6	2	4	0.0017		0.14 (0.02, 0.73)
nsp8	594	13	7	6	0.0016		0.32 (0.11, 0.98)
nsp9	339	15	9	6	0.0032		0.46 (0.17, 1.37)
nsp10	417	16	14	2	0.0028		1.99 (0.56, 12.67)
nsp12*	2795	122	69	53	0.0031		0.34 (0.24, 0.49)
ORF1b	8088	349	212	137	0.0031		0.42 (0.34, 0.52)
nsp13	1803	59	33	26	0.0024		0.37 (0.22, 0.63)
nsp14	1581	92	59	33	0.0042		0.48 (0.31, 0.74)
nsp15	1038	31	21	10	0.0021		0.57 (0.27, 1.26)
nsp16	894	45	30	15	0.0036		0.54 (0.29, 1.03)
S	3822	190	129	61	0.0036		0.6 (0.45, 0.82)
ORF3a	828	108	96	12	0.0094		2.29 (1.31, 4.4)
E	228	13	4	9	0.0041		0.15 (0.04, 0.47)
M	669	32	20	12	0.0034		0.51 (0.25, 1.08)
ORF6	186	10	8	2	0.0039		0.97 (0.24, 6.43)
ORF7a	366	41	34	7	0.0081		1.43 (0.67, 3.52)
ORF7b	132	8	8	0	0.0044		(0.93, )
ORF8	366	49	19	30	0.0096		0.17 (0.09, 0.3)
N	1260	145	106	39	0.0083		0.81 (0.56, 1.18)
ORF10	117	11	6	5	0.0068		0.32 (0.09, 1.09)
3UTR	229	74	-	-	0.0232		-
All coding regions	29260	1526	1009	517	0.0038		0.55 (0.49, 0.61)
Full genome	22903	1708	-	-	0.0041		-

All genome positions are relative to the Wuhan-Hu-1 reference sequence. iSNVs at the 18 highly shared sites and those identified from the synthetic controls are excluded, as are those in the poly-A tail (positions 29865 to 29903). The mean iSNVs per 100 sites column is the mean number in each gene over all 1390 sequenced genomes. Note that because of gene overlap and noncoding intergenic regions, the total number of iSNVs (1708) cannot be obtained as the sum of any column in this table, even if the rows for nonstructural proteins in ORF1ab are excluded.

*nsp12 overlaps the boundary between ORF1a and ORF1b.

Intergenic regions are excluded from this row.

## Within-host variant sites are phylogenetically associated

**Fig. 5:**
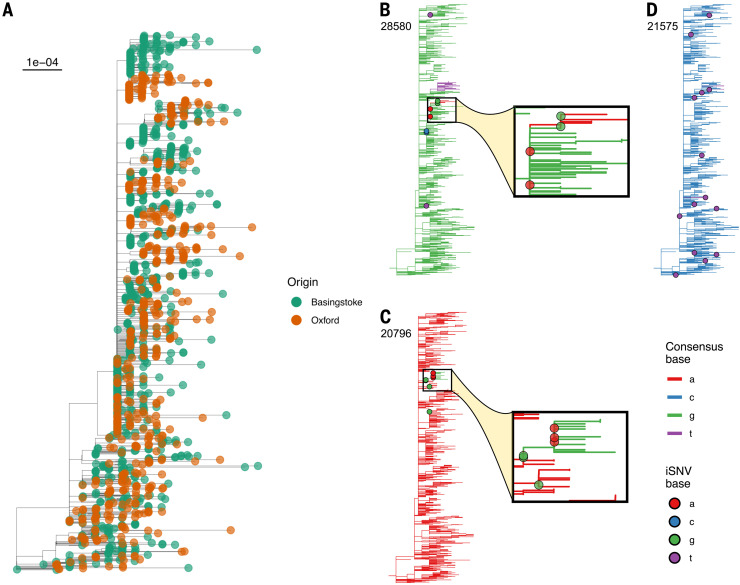
Consensus phylogeny of all isolates. In (**A**), tips are colored by sampling center (Oxford = orange; Basingstoke = green). The tree scale is in substitutions per site. (**B** to **D**) Distribution of samples with iSNVs at three loci. The genomic coordinate (with respect to the Wuhan-Hu-1 reference sequence) appears in the top left. Tree branches are colored by the consensus base at that position, and filled circles indicate iSNVs present at a minimum of 3% frequency in samples with depth of at least 100 at that position, and are colored by the most common minor variant present. For sites 28580 (B) and 20796 (C), an inset panel enlarges the section of the phylogeny where a consensus change is in close proximity to iSNVs with the relevant pair of nucleotides involved. The highlighted samples were prepared in separate batches and the patterns were not caused by contamination. (D) Variants at site 21575 (L5F) occurred in 14 samples but with no phylogenetic association with consensus changes at this site, which may represent independent emergence of this variant in multiple individuals. The phylogeny was constructed by maximum likelihood according to the robust procedure outlined by Morel *et al*. (*42*).

We sought to gain a better understanding of SARS-CoV-2 evolution and to determine whether iSNVs could be used to help resolve phylogenies and transmission clusters. For the 1390 genomes in our study, we constructed a phylogeny using the robust procedure outlined by (*42*) (Fig. 5A). Viral phylogenies are based on the consensus sequence for each sample, with branches indicating differences in the consensus sequence among samples. Given the inferred narrow transmission bottleneck size, we hypothesized that consensus changes on the phylogeny arise because of the emergence of within-host variants that either reach consensus within the individual in which they emerged or fail to reach consensus but are then transmitted and result in a consensus change in the recipient. In a sufficiently densely sampled population of infected individuals, we should therefore be able to observe a phylogenetic association between samples containing iSNVs with branches on the tree leading to a change in consensus at the same locus.

Of the 563 high-confidence iSNV sites, we identified 153 sites that were present in at least two samples and in which we also observed differences in the consensus among samples (SNPs). We call these sites iSNV-SNPs. We examined the proximity of tips with the iSNVs to the position of consensus changes (between the two most common bases at the site of the iSNV) on the phylogeny. A highly significant negative association (one-sided Mann-Whitney *U *test, *P *< 3 10^16^; fig. S6A) was found between the presence of an iSNV at a given site in a sample and the patristic distance to the nearest example of a consensus change at the same site; that is, intrahost variation clustered on the tree with branches supported by the same variant as consensus. When we tested sites where we had identified at least two iSNVs individually, six showed a significant association after Benjamini-Hochberg correction (*P *< 0.05), reducing to five if only one sample from each individual was included. Repeating this procedure on each of 1000 phylogenetic bootstrap replicates yielded a universally very strong association when taking sites across the whole genome (maximum *P* = 2.46 10^10^), whereas every bootstrapped tree had between one and nine significant iSNV-SNPs (median seven, IQR five to seven).

In Fig. 5B, we show the example of site 28580 (significant in 85.8% of bootstrap replicates), with the red clade representing change from the global consensus G to A (a nonsynonymous change D103N in N) and nearby iSNVs occurring both as minor As in the nodes ancestral to the change branch and as minor Gs in the branchs immediate descendants. Based on corresponding epidemiological data, this represents a health care-associated cluster with onward transmission to close contacts. In Fig. 5C, we give the further example of site 20796 (significant in 98.4% of bootstrap replicates), a synonymous substitution L6843 in ORF1a. Trees for the other significant sites after Benjamini-Hochberg correction are shown in fig. S7. Supporting this relationship between SNPs and iSNVs, we note that in the household transmission pairs that we examined, for the five consensus differences in which there was sufficient depth, all were within-host variant in one of the two individuals (Fig. 4B).

For the 261 iSNVs that were present in at least two individuals but never reached consensus, we analyzed the association with the phylogeny of each iSNV as a discrete trait using two statistics: the association index (*34*) and the mean patristic distance between iSNV tips. After adjustment for multiple testing, no sites showed a *P-*value <0.05 for a phylogeny-iSNV association for either statistic. Similarly, if we simply compared the distance to the nearest iSNV tip among iSNV and non-iSNV tips across all 261 iSNV sites, there was also no evidence of phylogenetic association (one-sided Mann-Whitney *U *test, *P * 1; fig. S6B). Nevertheless, some individual sites did show patterns suggestive of iSNV transmission, with diversity maintained after transmission (22 with *P *< 0.05 before adjustment for multiple testing for at least one of the two statistics; the nine with *P *< 0.025 are shown in fig. S7), suggesting that we may lack the power to statistically detect some associations. Among the 15 household transmission pairs, we observed only one iSNV shared in two individuals within the same household. This iSNV was specific to these two individuals in our dataset, demonstrating a likely example of transmitted viral diversity (Fig. 3B).

Taken together, our observations suggest that the transmission bottleneck can be wide enough to permit cotransmission of multiple genotypes in some instances but narrow enough that multiple variants do not persist after a small number of subsequent transmissions. In the cases in which transmission culminated in a consensus change on the phylogeny, these patterns were readily observable, but in most cases, we suggest that patterns of cotransmission were drowned out by the high proportion of iSNVs that failed to transmit or were transmitted but then lost. Analysis of transmission events over multiple generations is needed to fully elucidate these patterns.

Variants occurring repeatedly but without phylogenetic association could indicate sites under selection in distinct individuals (*43*). Of particular note are the variants that we observed at three sites in S: 21575 (L5F), 22899 (G446V), and 24198 (A879V), with G446V lying within the receptor-binding domain. The minor variant F5 was observed in 14 samples and represented SNPs in eight samples but did not have phylogenetic association in our iSNV-SNP analysis (*P* = 0.771 before multiple testing adjustment; Fig. 5D). This L5F mutation has been shown to increase infectivity in vitro (*44*) and has previously been identified as a potential site subject to selection (*45*). This variant has repeatedly been observed in global samples, including as minority variant, but appears to be increasing in frequency slowly if at all, suggesting that it is only advantageous within a small subset of individuals, with the variant either reverting in subsequent infections [as seen in HIV (*46*)] or failing to transmit at all. Similarly, we observed the minor variants V446 and V879 in four and six individuals, respectively. Both variants have previously been shown to reduce sensitivity to convalescent sera in vitro (*44*), and V446 strongly reduces binding of one of the antibodies (REGN10987) in the REGN-Cov2 antibody cocktail (*47*), suggesting that these may represent antibody escape mutations. We did not observe N501Y or E484K, both mutations of concern, in any of our samples (*48*).

## Concluding remarks

We uncovered a consistent and reproducible pattern of within-host SARS-CoV-2 diversity in a large dataset of >1000 individuals, with iSNV sites showing strong phylogenetic clustering patterns if they were also associated with a change in the consensus variant at the same site. However, most samples harbored few intrahost variants, and estimated transmission bottleneck sizes were very small, with maximum likelihood estimates between 1 and 8 among household transmission pairs. This means that if mutations do arise, they will be prone to loss at the point of transmission. The dense sampling and deep sequencing of SARS-CoV-2 has enabled us to witness evolution in action, with variants generated in one individual, if transmitted, leading to a change in consensus and fixation in subsequently infected individuals. This suggests that within-host variants could be used, at least in some instances, to help better resolve patterns of transmission in a background of low consensus diversity.

Our observations indicate that the within-host emergence of vaccine- and therapeutic-escape mutations is likely to be relatively rare, at least during early infection, when viral loads are high. However, even in the absence of vaccine or therapeutic selection pressure, potential host-adaptive mutations are observable with sufficient frequency that even a rare transmission event combined with narrow bottleneck size could result in rapid spread. Here, we identified 30 nonsynonymous minor variants in S that were present in multiple individuals (table S2). Two of these (G446V and A879V) have previously been shown to escape antibody binding (*44*), and a third, L5F, has been shown to increase viral infectivity (*44*). We suggest that commonly occurring iSNVs, along with variants known to affect transmissibility, severity of infection, or immune responses, should be investigated and monitored, particularly as vaccines and therapeutics are rolled out more widely.

The emergence of new variants of concern, including B.1.1.7, B.1.351, and P.1, underscores the need for continued vigilance. A leading hypothesis is that these variants, characterized by a large number of nonsynonymous mutations, originated within individuals with long durations of infection during which the virus was subject to prolonged immune pressure (*7*, *8*), and that this was potentially facilitated by the within-host emergence of deletions (*49*). However, the presence of multiple mutations on the same genetic background is not a necessary prerequisite for a new variant to be cause for concern. The single D614G S mutation spread globally after it emerged during the early stages of the pandemic, likely because of a transmission advantage (*50*). The potential for mutations including N439K and E484K, which may enable the virus to evade host-immune responses (*47*, *51*), to emerge on the highly transmissible B.1.1.7 background is also troubling, particularly as population immunity builds due to natural infection and vaccination.

Our work demonstrates that an essential requirement for incorporating intrahost variants in any analysis is an understanding of the observed intrahost diversity in the context of the methods used to produce the deep-sequencing data. Throughout this study, we aimed to minimize sequencing artifacts and sample contamination where possible. Moreover, our results emphasize the power of open data, large and rigorously controlled datasets, and the importance of integrating genomic, clinical, and epidemiological information to gain an in-depth understanding of SARS-CoV-2 as the pandemic unfolds.

## Materials and methods

### RNA extraction

Residual RNA from COVID-19 reverse transcription quantitative polymerase chain reaction (RT-qPCR)based testing was obtained from Oxford University Hospitals (hereafter Oxford), extracted on the QIASymphony platform with QIAsymphony DSP Virus/Pathogen Kit (QIAGEN), and from Basingstoke and North Hampshire Hospital (hereafter Basingstoke), extracted with one of the following: the Maxwell RSC Viral total nucleic acid kit (Promega), the Reliaprep blood gDNA miniprep system (Promega), or the Prepito NA body fluid kit (PerkinElmer). An internal extraction control was added to the lysis buffer before extraction to act as a control for extraction efficiency [genesig qRT-PCR kit, #Z-Path-2019-nCoV in Basingstoke, MS2 bacteriophage (*52*) in Oxford]. The #Z-Path-2019-nCoV control is a linear, synthetic RNA target based on sequence from the rat *ptprn2* gene, which has no sequence similarity with SARS-CoV-2 (GENESIG PrimerDesign, personal communication, 6 April 2020). The MS2 RNA likewise has no SARS-CoV-2 similarity (*52*). Neither control RNA interfered with sequencing.

### Targeted metagenomic sequencing

Samples with suspected epidemiological linkage, where this information was available before sequencing, were processed in different batches. Sequencing libraries were constructed from remnant volume of nucleic acid after clinical testing, ranging from 5 to 45 l (median 30 l) for each sample depending on the available amount of eluate. These volumes represented 1 to 15% of the original specimen (swab). Libraries were generated following the veSEQ protocol (*16*) with some modifications. Briefly, unique dual indexed (UDI) libraries for Illumina sequencing were constructed using the SMARTer Stranded Total RNA-Seq Kit v2 Pico Input Mammalian (Takara Bio) with no fragmentation of the RNA. An equal volume of library from each sample was pooled for capture. Size selection was performed on the captured pool to eliminate fragments shorter than 400 nucleotides (nt), which otherwise may be preferentially amplified and sequenced. Targeted enrichment of SARS-CoV-2 libraries in the pool was obtained through a custom xGen Lockdown Probes panel (IDT), using the SeqCap EZ Accessory Kits v2 and SeqCap Hybridization and Wash Kit (Roche) for hybridization of the probes and removal of unbound DNA. After 12 cycles of PCR for postcapture amplification, the final product was purified using Agencourt AMPure XP (Beckman Coulter). Sequencing was performed on the Illumina MiSeq (batches 1 and 2) or NovaSeq 6000 (batches 3 to 27) platform (Illumina) at the Oxford Genomics Centre, generating 150base pair (bp) or 250-bp paired-end reads.

### Quantification controls

A dilution series of in vitrotranscribed SARS-CoV-2 RNA [Twist Synthetic SARS-CoV-2 RNA Control 1 (MT007544.1), Twist Bioscience] was included in every capture pool of 90 samples starting from batch 3 and sequenced alongside the clinical samples. Control RNA was serially diluted into Universal Human Reference RNA (UHRR) to a final concentration of SARS-CoV-2 RNA of 500,000, 50,000, 5000, 500, 100, and 0 copies/reaction. From this, we produced a standard curve demonstrating linear association between viral load and read depth (fig. S1). For an experiment comparing iSNV presence with and without probe capture, we additionally sequenced two replicates of the Twist RNA control without capture, diluted into UHRR to give an expected concentration of 50,000 copies per reaction.

As an additional validation step, we compared iSNVs in resequenced controls with data for the stock RNA sequenced and provided by the manufacturer (Twist Bioscience). Six well-defined iSNVs, which were present in the manufacturers data and presumably arose during in vitro transcription, were also recovered by our protocol (fig. S8). In addition, we identified 112 sites that appeared vulnerable to low-frequency intrahost variation in vitro (table S3), possibly as a result of structural variation along the genome or interaction with the sequencing protocol. We blacklisted vulnerable sites from further analysis.

### In-run controls

In addition to the synthetic RNA standards described above, each batch included a non-SARS-CoV-2 in-run control consisting of purified, in vitrotranscribed HIV RNA from clone p92BR025.8 obtained from the National Institute for Biological Standards and Control (*53*). For batches 1 and 2, which were sequenced before synthetic RNA became available, we included negative buffer controls. As additional negative controls, we sequenced six matched clinical samples from nonCOVID-19 patients distributed across different sequencing runs, and none contained any SARS-CoV-2 reads.

### Minimizing risk of index misassignment

All samples had UDI to prevent cross-detection of reads in the same pool. The in-run HIV RNA controls were used to estimate index misassignment because this provided a sequence-distinct source of RNA: <3 SARS-CoV-2 reads were detected in any HIV control (median 0), and <10 HIV reads were detected in any SARS-CoV-2 control (median 0), suggesting that index misassignment, if present, occurred at extremely low levels.

### Bioinformatics processing

Demultiplexed sequence read pairs were classified by Kraken version 2 (*54*) using a custom database containing the human genome (GRCh38 build) and the full RefSeq set of bacterial and viral genomes (pulled May 2020). Sequences identified as either human or bacterial were removed using filter_keep_reads.py from the Castanet (*17*) workflow (*55*). Remaining reads, composed of viral and unclassified reads, were trimmed in two stages: first to remove the random hexamer primers from the forward read and SMARTer TSO from the reverse read, and then to remove Illumina adapter sequences using Trimmomatic version 0.36 (*56*), with the ILLUMINACLIP options set to 2:10:7:1:true MINLEN:80. Trimmed reads were mapped to the SARS-CoV-2 RefSeq genome of isolate Wuhan-Hu-1 (NC_045512.2) using *shiver* (*57*) version 1.5.7, with either smalt (*58*) or bowtie2 (*59*) as the mapper. Both mappers generated comparable results, and smalt was used for the final analysis. Only properly paired reads with insert size <2000 and with at least 70% sequence identity to the reference were retained. For analysis of consensus genomes, consensus calls required a minimum of two uniquely mapped (deduplicated) reads per position, equivalent to >15 raw reads per position. Analysis of within-host diversity was restricted only to positions with minimum raw depth of 100, except when examining diversity within presumed recipients of transmissions in the bottleneck analysis. MAFs were computed at every position using shiver (*57*) (tools/AnalysePileup.py), with the default settings of no BAQ and maximum pileup depth of 1000000. Lineages were assigned by the Pangolin web server (*60*) using the determined consensus genome for each sequenced sample.

### Alignment

Oxford and Basingstoke samples were selected if the consensus sequence (inferred from unique mapped reads) consisted of no more than 25% N characters. As an alignment to the reference sequence was already performed in *shiver*, no further alignment was necessary. To place these data into the global phylogenetic context and to help resolve ancestry, a collection of non-UK consensus sequences from the GISAID database (*61*) were included in the set of sequences to be aligned. All GISAID (*62*) sequences were downloaded from the database on 26 April 2020 and filtered to remove sequences that were <29,800 base pairs in length, had >1% Ns, or were from the United Kingdom. The remaining sequences were clustered using CD-HIT-EST (*63*) using a similarity threshold of 0.995, and then one sequence per cluster picked. The resulting set, along with the reference genome Wuhan-Hu-1 (RefSeq ID NC_045512), were aligned using MAFFT (*64*), with some manual improvement of the algorithmic alignment and removal of problematic sequences performed as a postprocessing step. Indels with respect to Wuhan-Hu-1 in both the Oxford and/or Basingstoke and GISAID alignments were deleted, resulting in two alignments of 29,903 nucleotides that could be readily combined.

### Demonstration of the effect of read down-sampling

To demonstrate the effect of read depth on estimated iSNV counts, we selected the 30 samples with the highest total number of mapped reads, chose a variety of down-sampling fractions for each, and removed all but that proportion of called bases from consideration. We then determined, for each sample and fraction, the number of iSNVs that would be identified at a threshold of 3% MAF at a minimum depth of 100 if only that fraction of called bases were available to us.

### Transmission bottleneck analysis

Sixteen potential transmission pairs were identified by shared address (household) and first positive sample within 2 weeks. If samples from the two individuals in the household differed by fewer than three consensus differences (15 households), direct transmission was assumed. Apart from one genome position in household 6 and one in household 12, all sites associated with a consensus difference within a household were within-host variable in at least one member of the household pair, lending support to assumption of direct transmission (the exceptions are associated with low-read samples). Household 15 had six consensus differences and was therefore excluded from our bottleneck analysis, although we note that for all six positions, the site was within-host variable in one or other individual. This pattern is inconsistent with direct transmission but may represent transmission from a common source. When the first samples for each individual in the household were >1 week apart, we assumed that the earlier sampled individual was the source; otherwise, we considered both possible directions of transmission. If individuals had more than one sample or replicate sequences from the same sample, then we used the sample and/or replicate with the highest number of mapped reads.

Bottleneck size was calculated using the exact beta-binomial method described in (*28*). Because most samples in the analysis had <50,000 mapped reads, we considered all sites in the genome, including sites in the 3 and 5 UTR, but excluding the poly-A tail (positions 29865 to 29903), the 18 highly shared sites, and those identified from the synthetic controls. All sites with >3% MAF and >100 reads in the assumed source individual were used in the analysis. In the recipient, all reads at these sites were considered, with an error threshold of 0.5% MAF. Following (*28*), 95% confidence intervals (CIs) were calculated using a likelihood ratio test. No estimate was recorded for household 8 because there were no identified iSNVs >3% in the source.

### Calculation of dN/dS

The total number of synonymous and nonsynonymous substitutions in the SARS-CoV-2 genome was estimated using the first method of (*65*) applied to the coding regions of the Wuhan-Hu-1 reference sequence. Overlapping reading frames were accounted for such that a substitution was considered nonsynonymous overall if it was nonsynonymous in either frame.

We took two approaches to this calculation, first by counting all iSNVs individually, and second by counting only unique iSNVs. In the latter case, where we detected iSNVs with different base changes at the same position, we included only the most frequent. The results of the former are the basis for Table 1, whereas those of the latter appear in table S5.

The *dN/dS* ratio for iSNVs over a genomic region *G* was then calculated as follows:

$$\frac{{}_{pG}i_{p}^{N}}{T_{G}^{N}}/\frac{{}_{pG}i_{p}^{S}}{T_{G}^{S}}$$

where $i_{p}^{N}$ is the fraction of iSNVs at *p* that are nonsynonymous, or 0 if there are no iSNVs at *p*; $T_{G}^{N}$ is the total number of potential nonsynonymous substitutions in *G*; and the denominator replaces *N* with *S* to represent synonymous substitutions. The 95% CIs for these estimates were obtained using the likelihood ratio test.

### Phylogenetics

Phylogenetic reconstruction was performed on the alignment consisting of the 1390 consensus sequences, along with the GISAID set and the Wuhan-Hu-1 reference sequence. We followed the recommendations of Morel *et al*. (*42*), in which 100 separate maximum likelihood phylogenies were generated using RAxML-NG (*66*) and the GTR+G substitution model, such that each reconstruction used a different random starting parsimony tree. The final phylogeny was then obtained from this set using majority rule. This final tree was rooted with respect to the reference sequence, and then that and all GISAID isolates were pruned.

To identify homoplasic sites, we selected sites that changed state more than once along the tree after inferring the states at internal nodes using ancestral state reconstruction as implemented in ClonalFrameML (*67*) and rooting the tree using the reference genome NC_045512.

The recommendations of Morel *et al*. do not easily lend themselves to fast bootstrapping, so to explore phylogenetic uncertainty, we performed an additional phylogenetic reconstruction on the same alignment using the ultrafast bootstrap procedure in IQ-TREE (*68*). A total of 1000 bootstrap replicates were used.

### Phylogenetic association of iSNVs and SNPs

Where an iSNV corresponded to a consensus SNP (by the base pair involved, not simply the site), we performed ancestral state reconstruction on the consensus trees using ClonalFrameML (*67*) to identify all branches upon which that substitution was involved. Tips derived from the same clinical sample were then pruned until only one (the one with the highest overall depth) remained. Then, for each tip in the tree, we calculated the patristic distance from that tip to the midpoint of the closest one of these branches and used a one-tailed Mann-Whitney *U *test to test for association between the iSNV existing in a sample and this distance. Multiple testing was controlled for using the Benjamini-Hochberg adjustment. As a sensitivity analysis, this was repeated such that all but one tip per infected individual, rather than per clinical sample, were pruned. These analyses were done both on an individual site level and across all sites of interest.

To confirm that the associations that we observed here were unaffected by phylogenetic uncertainty, we used the set of 1000 IQ-TREE bootstraps. We repeated the Mann-Whitney *U* tests above for each of these 1000 trees.

### Phylogenetic association of iSNVs at consensus invariant positions

For the remaining iSNVs, we calculated the extent of association with the consensus phylogeny by treating the presence of an iSNV as a discrete character and calculating the association index and the mean patristic distance between iSNV tips. Once again, the consensus tree was pruned such that tips corresponding to samples with read depth <100 at the position and all but one tip coming from the same individual were removed. A null distribution was generated by permuting the tip labels of this tree 10,000 times, and a one-sided permutation test *P*-value was calculated. Multiple testing was adjusted for as above. In addition, for each tip in the phylogeny at each site of interest, we calculated the minimum patristic distance to a different tip corresponding to an iSNV and used the Mann-Whitney *U *test again to compare the distribution of these distances between iSNV and non-iSNV tips.

## Supplementary Materials

science.sciencemag.org/content/372/6539/eabg0821/suppl/DC1 Figs. S1 to S8 Tables S1 to S5 List of OVSG members List of COG-UK consortium names and affiliations MDAR Reproducibility Checklist

## Appendix

View/request a protocol for this paper from *Bio-protocol*.

## References

[1] A. Rambaut, E. C. Holmes, . OToole, V. Hill, J. T. McCrone, C. Ruis, L. du Plessis, O. G. Pybus, A dynamic nomenclature proposal for SARS-CoV-2 lineages to assist genomic epidemiology. Nat. Microbiol. 5, 1403 1407 (2020). 10.1038/s41564-020-0770-5 32669681 PMC7610519

[2] J. Hadfield, C. Megill, S. M. Bell, J. Huddleston, B. Potter, C. Callender, P. Sagulenko, T. Bedford, R. A. Neher, Nextstrain: Real-time tracking of pathogen evolution. Bioinformatics 34, 4121 4123 (2018). 10.1093/bioinformatics/bty407 29790939 PMC6247931

[3] J. Lu, L. du Plessis, Z. Liu, V. Hill, M. Kang, H. Lin, J. Sun, S. Franois, M. U. G. Kraemer, N. R. Faria, J. T. McCrone, J. Peng, Q. Xiong, R. Yuan, L. Zeng, P. Zhou, C. Liang, L. Yi, J. Liu, J. Xiao, J. Hu, T. Liu, W. Ma, W. Li, J. Su, H. Zheng, B. Peng, S. Fang, W. Su, K. Li, R. Sun, R. Bai, X. Tang, M. Liang, J. Quick, T. Song, A. Rambaut, N. Loman, J. Raghwani, O. G. Pybus, C. Ke, Genomic epidemiology of SARS-CoV-2 in Guangdong Province, China. Cell 181, 997 1003.e9 (2020). 10.1016/j.cell.2020.04.023 32359424 PMC7192124

[4] B. Korber, W. M. Fischer, S. Gnanakaran, H. Yoon, J. Theiler, W. Abfalterer, N. Hengartner, E. E. Giorgi, T. Bhattacharya, B. Foley, K. M. Hastie, M. D. Parker, D. G. Partridge, C. M. Evans, T. M. Freeman, T. I. de Silva, C. McDanal, L. G. Perez, H. Tang, A. Moon-Walker, S. P. Whelan, C. C. LaBranche, E. O. Saphire, D. C. Montefiori, A. Angyal, R. L. Brown, L. Carrilero, L. R. Green, D. C. Groves, K. J. Johnson, A. J. Keeley, B. B. Lindsey, P. J. Parsons, M. Raza, S. Rowland-Jones, N. Smith, R. M. Tucker, D. Wang, M. D. Wyles; Sheffield COVID-19 Genomics Group, Tracking changes in SARS-CoV-2 spike: Evidence that D614G increases infectivity of the COVID-19 virus. Cell 182, 812 827.e19 (2020). 10.1016/j.cell.2020.06.043 32697968 PMC7332439

[5] E. Volz, V. Hill, J. T. McCrone, A. Price, D. Jorgensen, . OToole, J. Southgate, R. Johnson, B. Jackson, F. F. Nascimento, S. M. Rey, S. M. Nicholls, R. M. Colquhoun, A. da Silva Filipe, J. Shepherd, D. J. Pascall, R. Shah, N. Jesudason, K. Li, R. Jarrett, N. Pacchiarini, M. Bull, L. Geidelberg, I. Siveroni, I. Goodfellow, N. J. Loman, O. G. Pybus, D. L. Robertson, E. C. Thomson, A. Rambaut, T. R. Connor, C. Koshy, E. Wise, N. Cortes, J. Lynch, S. Kidd, M. Mori, D. J. Fairley, T. Curran, J. P. McKenna, H. Adams, C. Fraser, T. Golubchik, D. Bonsall, C. Moore, S. L. Caddy, F. A. Khokhar, M. Wantoch, N. Reynolds, B. Warne, J. Maksimovic, K. Spellman, K. McCluggage, M. John, R. Beer, S. Afifi, S. Morgan, A. Marchbank, A. Price, C. Kitchen, H. Gulliver, I. Merrick, J. Southgate, M. Guest, R. Munn, T. Workman, T. R. Connor, W. Fuller, C. Bresner, L. B. Snell, T. Charalampous, G. Nebbia, R. Batra, J. Edgeworth, S. C. Robson, A. Beckett, K. F. Loveson, D. M. Aanensen, A. P. Underwood, C. A. Yeats, K. Abudahab, B. E. W. Taylor, M. Menegazzo, G. Clark, W. Smith, M. Khakh, V. M. Fleming, M. M. Lister, H. C. Howson-Wells, L. Berry, T. Boswell, A. Joseph, I. Willingham, P. Bird, T. Helmer, K. Fallon, C. Holmes, J. Tang, V. Raviprakash, S. Campbell, N. Sheriff, M. W. Loose, N. Holmes, C. Moore, M. Carlile, V. Wright, F. Sang, J. Debebe, F. Coll, A. W. Signell, G. Betancor, H. D. Wilson, T. Feltwell, C. J. Houldcroft, S. Eldirdiri, A. Kenyon, T. Davis, O. Pybus, L. du Plessis, A. Zarebski, J. Raghwani, M. Kraemer, S. Francois, S. Attwood, T. Vasylyeva, M. E. Torok, W. L. Hamilton, I. G. Goodfellow, G. Hall, A. S. Jahun, Y. Chaudhry, M. Hosmillo, M. L. Pinckert, I. Georgana, A. Yakovleva, L. W. Meredith, S. Moses, H. Lowe, F. Ryan, C. L. Fisher, A. R. Awan, J. Boyes, J. Breuer, K. A. Harris, J. R. Brown, D. Shah, L. Atkinson, J. C. D. Lee, A. Alcolea-Medina, N. Moore, N. Cortes, R. Williams, M. R. Chapman, L. J. Levett, J. Heaney, D. L. Smith, M. Bashton, G. R. Young, J. Allan, J. Loh, P. A. Randell, A. Cox, P. Madona, A. Holmes, F. Bolt, J. Price, S. Mookerjee, A. Rowan, G. P. Taylor, M. Ragonnet-Cronin, F. F. Nascimento, D. Jorgensen, I. Siveroni, R. Johnson, O. Boyd, L. Geidelberg, E. M. Volz, K. Brunker, K. L. Smollett, N. J. Loman, J. Quick, C. McMurray, J. Stockton, S. Nicholls, W. Rowe, R. Poplawski, R. T. Martinez-Nunez, J. Mason, T. I. Robinson, E. OToole, J. Watts, C. Breen, A. Cowell, C. Ludden, G. Sluga, N. W. Machin, S. S. Y. Ahmad, R. P. George, F. Halstead, V. Sivaprakasam, E. C. Thomson, J. G. Shepherd, P. Asamaphan, M. O. Niebel, K. K. Li, R. N. Shah, N. G. Jesudason, Y. A. Parr, L. Tong, A. Broos, D. Mair, J. Nichols, S. N. Carmichael, K. Nomikou, E. Aranday-Cortes, N. Johnson, I. Starinskij, A. da Silva Filipe, D. L. Robertson, R. J. Orton, J. Hughes, S. Vattipally, J. B. Singer, A. D. Hale, L. R. Macfarlane-Smith, K. L. Harper, Y. Taha, B. A. I. Payne, S. Burton-Fanning, S. Waugh, J. Collins, G. Eltringham, K. E. Templeton, M. P. McHugh, R. Dewar, E. Wastenge, S. Dervisevic, R. Stanley, R. Prakash, C. Stuart, N. Elumogo, D. K. Sethi, E. J. Meader, L. J. Coupland, W. Potter, C. Graham, E. Barton, D. Padgett, G. Scott, E. Swindells, J. Greenaway, A. Nelson, W. C. Yew, P. C. Resende Silva, M. Andersson, R. Shaw, T. Peto, A. Justice, D. Eyre, D. Crooke, S. Hoosdally, T. J. Sloan, N. Duckworth, S. Walsh, A. J. Chauhan, S. Glaysher, K. Bicknell, S. Wyllie, E. Butcher, S. Elliott, A. Lloyd, R. Impey, N. Levene, L. Monaghan, D. T. Bradley, E. Allara, C. Pearson, P. Muir, I. B. Vipond, R. Hopes, H. M. Pymont, S. Hutchings, M. D. Curran, S. Parmar, A. Lackenby, T. Mbisa, S. Platt, S. Miah, D. Bibby, C. Manso, J. Hubb, M. Chand, G. Dabrera, M. Ramsay, D. Bradshaw, A. Thornton, R. Myers, U. Schaefer, N. Groves, E. Gallagher, D. Lee, D. Williams, N. Ellaby, I. Harrison, H. Hartman, N. Manesis, V. Patel, C. Bishop, V. Chalker, H. Osman, A. Bosworth, E. Robinson, M. T. G. Holden, S. Shaaban, A. Birchley, A. Adams, A. Davies, A. Gaskin, A. Plimmer, B. Gatica-Wilcox, C. McKerr, C. Moore, C. Williams, D. Heyburn, E. De Lacy, E. Hilvers, F. Downing, G. Shankar, H. Jones, H. Asad, J. Coombes, J. Watkins, J. M. Evans, L. Fina, L. Gifford, L. Gilbert, L. Graham, M. Perry, M. Morgan, M. Bull, M. Cronin, N. Pacchiarini, N. Craine, R. Jones, R. Howe, S. Corden, S. Rey, S. Kumziene-Summerhayes, S. Taylor, S. Cottrell, S. Jones, S. Edwards, J. OGrady, A. J. Page, J. Wain, M. A. Webber, A. E. Mather, D. J. Baker, S. Rudder, M. Yasir, N. M. Thomson, A. Aydin, A. P. Tedim, G. L. Kay, A. J. Trotter, R. A. J. Gilroy, N.-F. Alikhan, L. de Oliveira Martins, T. Le-Viet, L. Meadows, A. Kolyva, M. Diaz, A. Bell, A. V. Gutierrez, I. G. Charles, E. M. Adriaenssens, R. A. Kingsley, A. Casey, D. A. Simpson, Z. Molnar, T. Thompson, E. Acheson, J. A. H. Masoli, B. A. Knight, A. Hattersley, S. Ellard, C. Auckland, T. W. Mahungu, D. Irish-Tavares, T. Haque, Y. Bourgeois, G. P. Scarlett, D. G. Partridge, M. Raza, C. Evans, K. Johnson, S. Liggett, P. Baker, S. Essex, R. A. Lyons, L. G. Caller, S. Castellano, R. J. Williams, M. Kristiansen, S. Roy, C. A. Williams, P. L. Dyal, H. J. Tutill, Y. N. Panchbhaya, L. M. Forrest, P. Niola, J. Findlay, T. T. Brooks, A. Gavriil, L. Mestek-Boukhibar, S. Weeks, S. Pandey, L. Berry, K. Jones, A. Richter, A. Beggs, C. P. Smith, G. Bucca, A. R. Hesketh, E. M. Harrison, S. J. Peacock, S. Palmer, C. M. Churcher, K. L. Bellis, S. T. Girgis, P. Naydenova, B. Blane, S. Sridhar, C. Ruis, S. Forrest, C. Cormie, H. K. Gill, J. Dias, E. E. Higginson, M. Maes, J. Young, L. M. Kermack, N. F. Hadjirin, D. Aggarwal, L. Griffith, T. Swingler, R. K. Davidson, A. Rambaut, T. Williams, C. E. Balcazar, M. D. Gallagher, . OToole, S. Rooke, B. Jackson, R. Colquhoun, J. Ashworth, V. Hill, J. T. McCrone, E. Scher, X. Yu, K. A. Williamson, T. D. Stanton, S. L. Michell, C. M. Bewshea, B. Temperton, M. L. Michelsen, J. Warwick-Dugdale, R. Manley, A. Farbos, J. W. Harrison, C. M. Sambles, D. J. Studholme, A. R. Jeffries, A. C. Darby, J. A. Hiscox, S. Paterson, M. Iturriza-Gomara, K. A. Jackson, A. O. Lucaci, E. E. Vamos, M. Hughes, L. Rainbow, R. Eccles, C. Nelson, M. Whitehead, L. Turtle, S. T. Haldenby, R. Gregory, M. Gemmell, D. Kwiatkowski, T. I. de Silva, N. Smith, A. Angyal, B. B. Lindsey, D. C. Groves, L. R. Green, D. Wang, T. M. Freeman, M. D. Parker, A. J. Keeley, P. J. Parsons, R. M. Tucker, R. Brown, M. Wyles, C. Constantinidou, M. Unnikrishnan, S. Ott, J. K. J. Cheng, H. E. Bridgewater, L. R. Frost, G. Taylor-Joyce, R. Stark, L. Baxter, M. T. Alam, P. E. Brown, P. C. McClure, J. G. Chappell, T. Tsoleridis, J. Ball, D. Gramatopoulos, D. Buck, J. A. Todd, A. Green, A. Trebes, G. MacIntyre-Cockett, M. de Cesare, C. Langford, A. Alderton, R. Amato, S. Goncalves, D. K. Jackson, I. Johnston, J. Sillitoe, S. Palmer, M. Lawniczak, M. Berriman, J. Danesh, R. Livett, L. Shirley, B. Farr, M. Quail, S. Thurston, N. Park, E. Betteridge, D. Weldon, S. Goodwin, R. Nelson, C. Beaver, L. Letchford, D. A. Jackson, L. Foulser, L. McMinn, L. Prestwood, S. Kay, L. Kane, M. J. Dorman, I. Martincorena, C. Puethe, J.-P. Keatley, G. Tonkin-Hill, C. Smith, D. Jamrozy, M. A. Beale, M. Patel, C. Ariani, M. Spencer-Chapman, E. Drury, S. Lo, S. Rajatileka, C. Scott, K. James, S. K. Buddenborg, D. J. Berger, G. Patel, M. V. Garcia-Casado, T. Dibling, S. McGuigan, H. A. Rogers, A. D. Hunter, E. Souster, A. S. Neaverson; COG-UK Consortium, Evaluating the effects of SARS-CoV-2 spike mutation D614G on transmissibility and pathogenicity. Cell 184, 64 75.e11 (2021). 10.1016/j.cell.2020.11.020 33275900 PMC7674007

[6] Y. J. Hou, S. Chiba, P. Halfmann, C. Ehre, M. Kuroda, K. H. Dinnon 3rd, S. R. Leist, A. Schfer, N. Nakajima, K. Takahashi, R. E. Lee, T. M. Mascenik, R. Graham, C. E. Edwards, L. V. Tse, K. Okuda, A. J. Markmann, L. Bartelt, A. de Silva, D. M. Margolis, R. C. Boucher, S. H. Randell, T. Suzuki, L. E. Gralinski, Y. Kawaoka, R. S. Baric, SARS-CoV-2 D614G variant exhibits efficient replication ex vivo and transmission in vivo. Science 370, 1464 1468 (2020). 33184236 10.1126/science.abe8499 PMC7775736

[7] A. Rambaut, N. Loman, O. Pybus, W. Barclay, J. Barrett, A. Carabelli, T. Connor, T. Peacock, D. L. Robertson, E. Volz, on behalf of COVID-19 Genomics Consortium UK (CoG-UK), Preliminary genomic characterisation of an emergent SARS-CoV-2 lineage in the UK defined by a novel set of spike mutations (2020); https://virological.org/t/preliminary-genomic-characterisation-of-an-emergent-sars-cov-2-lineage-in-the-uk-defined-by-a-novel-set-of-spike-mutations/563.

[8] S. A. Kemp, W. T. Harvey, S. Lytras, The COVID-19 Genomics UK (COG-UK) consortium, A. M. Carabelli, D. L. Robertson, R. K. Gupta, Recurrent emergence and transmission of a SARS-CoV-2 Spike deletion H69/V70. bioRxiv 422555 [Preprint] 9 February 2021. 10.1101/2020.12.14.422555.

[9] E. Volz, S. Mishra, M. Chand, J. C. Barrett, R. Johnson, L. Geidelberg, W. R. Hinsley, D. J. Laydon, G. Dabrera, . OToole, R. Amato, M. Ragonnet-Cronin, I. Harrison, B. Jackson, C. V. Ariani, O. Boyd, N. J. Loman, J. T. McCrone, S. Gonalves, D. Jorgensen, R. Myers, V. Hill, D. K. Jackson, K. Gaythorpe, N. Groves, J. Sillitoe, D. P. Kwiatkowski, The COVID-19 Genomics UK (COG-UK) consortium, S. Flaxman, O. Ratmann, S. Bhatt, S. Hopkins, A. Gandy, A. Rambaut, N. M. Ferguson, Transmission of SARS-CoV-2 lineage B.1.1.7 in England: Insights from linking epidemiological and genetic data. medRxiv 20249034 [Preprint]. 4 January 2021. 10.1101/2020.12.30.20249034.

[10] H. Tegally, E. Wilkinson, M. Giovanetti, A. Iranzadeh, V. Fonseca, J. Giandhari, D. Doolabh, S. Pillay, E. J. San, N. Msomi, K. Mlisana, A. von Gottberg, S. Walaza, M. Allam, A. Ismail, T. Mohale, A. J. Glass, S. Engelbrecht, G. Van Zyl, W. Preiser, F. Petruccione, A. Sigal, D. Hardie, G. Marais, M. Hsiao, S. Korsman, M.-A. Davies, L. Tyers, I. Mudau, D. York, C. Maslo, D. Goedhals, S. Abrahams, O. Laguda-Akingba, A. Alisoltani-Dehkordi, A. Godzik, C. K. Wibmer, B. T. Sewell, J. Loureno, L. C. J. Alcantara, S. L. Kosakovsky Pond, S. Weaver, D. Martin, R. J. Lessells, J. N. Bhiman, C. Williamson, T. de Oliveira, Emergence and rapid spread of a new severe acute respiratory syndrome-related coronavirus 2 (SARS-CoV-2) lineage with multiple spike mutations in South Africa. medRxiv 20248640 [Preprint]. 22 December 2020. 10.1101/2020.12.21.20248640.

[11] N. R. Faria, I. M. Claro, D. Candido, L. A. Moyses Franco, P. S. Andrade, T. M. Coletti, C. A. Silva, F. C. Sales, E. R. Manuli, R. S. Aguiar, N. Gaburo, C. da C. Camilo, N. A. Fraiji, M. A. Esashika Crispin, M. do Perpetuao, A. Rambaut, N. Loman, O. G. Pybus, E. C. Sabino, on behalf of CADDE Genomic Network, Genomic characterisation of an emergent SARS-CoV-2 lineage in Manaus: preliminary findings (2021); https://virological.org/t/genomic-characterisation-of-an-emergent-sars-cov-2-lineage-in-manaus-preliminary-findings/586).

[12] C. K. Wibmer, F. Ayres, T. Hermanus, M. Madzivhandila, P. Kgagudi, B. E. Lambson, M. Vermeulen, K. van den Berg, T. Rossouw, M. Boswell, V. Ueckermann, S. Meiring, A. von Gottberg, C. Cohen, L. Morris, J. N. Bhiman, P. L. Moore, SARS-CoV-2 501Y.V2 escapes neutralization by South African COVID-19 donor plasma. bioRxiv 427166 [Preprint]. 1 March 2021. 10.1101/2021.01.18.427166. 33654292

[13] S. Cele, I. Gazy, L. Jackson, S.-H. Hwa, H. Tegally, G. Lustig, J. Giandhari, S. Pillay, E. Wilkinson, Y. Naidoo, F. Karim, Y. Ganga, K. Khan, A. B. Balazs, B. I. Gosnell, W. Hanekom, M.-Y. S. Moosa, NGS-SA, COMMIT-KZN Team, R. J. Lessells, T. de Oliveira, A. Sigal, Escape of SARS-CoV-2 501Y.V2 variants from neutralization by convalescent plasma. medRxiv 21250224 [Preprint]. 27 February 2021. 10.1101/2021.01.26.21250224.

[14] K. Wu, A. P. Werner, J. I. Moliva, M. Koch, A. Choi, G. B. E. Stewart-Jones, H. Bennett, S. Boyoglu-Barnum, W. Shi, B. S. Graham, A. Carfi, K. S. Corbett, R. A. Seder, D. K. Edwards, mRNA-1273 vaccine induces neutralizing antibodies against spike mutants from global SARS-CoV-2 variants. bioRxiv 427948 [Preprint]. 25 January 2021. 10.1101/2021.01.25.427948.

[15] L. du Plessis, J. T. McCrone, A. E. Zarebski, V. Hill, C. Ruis, B. Gutierrez, J. Raghwani, J. Ashworth, R. Colquhoun, T. R. Connor, N. R. Faria, B. Jackson, N. J. Loman, . OToole, S. M. Nicholls, K. V. Parag, E. Scher, T. I. Vasylyeva, E. M. Volz, A. Watts, I. I. Bogoch, K. Khan, COVID-19 Genomics UK (COG-UK) Consortium, D. M. Aanensen, M. U. G. Kraemer, A. Rambaut, O. G. Pybus, Establishment and lineage dynamics of the SARS-CoV-2 epidemic in the UK. Science 10.1126/science.abf2946 (2021). 10.1126/science.abf2946 PMC7877493 33419936

[16] D. Bonsall, T. Golubchik, M. de Cesare, M. Limbada, B. Kosloff, G. MacIntyre-Cockett, M. Hall, C. Wymant, M. A. Ansari, L. Abeler-Drner, A. Schaap, A. Brown, E. Barnes, E. Piwowar-Manning, S. Eshleman, E. Wilson, L. Emel, R. Hayes, S. Fidler, H. Ayles, R. Bowden, C. Fraser; HPTN 071 (PopART) Team, A comprehensive genomics solution for HIV surveillance and clinical monitoring in low-income settings. J. Clin. Microbiol. 58, e00382-20 (2020). 10.1128/JCM.00382-20 32669382 PMC7512176

[17] C. Goh, T. Golubchik, A. Anzari, M. de Cesare, A. Trebes, I. Elliott, D. Bonsall, P. Piazza, A. Brown, H. Slawinski, N. Martin, S. Defres, M. J. Griffiths, J. E. Bray, M. C. Maiden, P. Hutton, C. J. Hinds, T. Solomon, E. Barnes, A. J. Pollard, M. Sadarangani, J. C. Knight, R. Bowden, Targeted metagenomic sequencing enhances the identification of pathogens associated with acute infection. bioRxiv 716902 [Preprint]. 28 July 2019. 10.1101/716902.

[18] G.-L. Lin, T. Golubchik, S. Drysdale, D. OConnor, K. Jefferies, A. Brown, M. de Cesare, D. Bonsall, M. A. Ansari, J. Aerssens, L. Bont, P. Openshaw, F. Martinn-Torres, R. Bowden, A. J. Pollard, H. Nair, H. Campbell, S. Cunningham, P. Beutels, L. Bont, J. Wildenbeest, A. Pollard, C. Butler, M. Snape, S. Drysdale, G.-L. Lin, D. OConnor, E. Clutterbuck, K. Jefferies, J. McGinley, P. Openshaw, R. Thwaites, D. Wiseman, F. Martinon-Torres, A. Gmez-Carballa, T. Heikkinen, A. Meijer, T. K. Fischer, M. van den Berge, C. Giaquinto, M. Abram, K. Swanson, A. Leach, C. Demont, S. Gallichan, J. Aerssens, D. ner, B. Rosen, E. Molero, H. Nair, H. Campbell, S. Cunningham, P. Beutels, L. Bont, J. Wildenbeest, A. Pollard, C. Butler, M. Snape, S. Drysdale, G.-L. Lin, D. OConnor, E. Clutterbuck, K. Jefferies, J. McGinley, P. Openshaw, R. Thwaites, D. Wiseman, F. Martinon-Torres, A. Gmez-Carballa, T. Heikkinen, A. Meijer, T. K. Fischer, M. van den Berge, C. Giaquinto, M. Abram, K. Swanson, A. Leach, C. Demont, S. Gallichan, J. Aerssens, D. ner, B. Rosen, E. Molero; RESCEU Investigators, Simultaneous viral whole-genome sequencing and differential expression profiling in respiratory syncytial virus infection of infants. J. Infect. Dis. 222, S666 S671 (2020). 10.1093/infdis/jiaa448 32702120

[19] D. Bonsall, M. A. Ansari, C. Ip, A. Trebes, A. Brown, P. Klenerman, D. Buck, P. Piazza, E. Barnes, R. Bowden; STOP-HCV Consortium, ve-SEQ: Robust, unbiased enrichment for streamlined detection and whole-genome sequencing of HCV and other highly diverse pathogens. F1000Res. 4, 1062 (2015). 10.12688/f1000research.7111.1 27092241 PMC4821293

[20] S. M. Kissler, J. R. Fauver, C. Mack, S. W. Olesen, C. Tai, K. Y. Shiue, C. C. Kalinich, S. Jednak, I. M. Ott, C. B. F. Vogels, J. Wohlgemuth, J. Weisberger, J. DiFiori, D. J. Anderson, J. Mancell, D. D. Ho, N. D. Grubaugh, Y. H. Grad, SARS-CoV-2 viral dynamics in acute infections. medRxiv 20217042 [Preprint]. 1 December 2020. 10.1101/2020.10.21.20217042.

[21] L. Ferretti, A. Ledda, C. Wymant, L. Zhao, V. Ledda, L. A. Dorner, M. Kendall, A. Nurtay, H.-Y. Cheng, T.-C. Ng, H.-H. Lin, R. Hinch, J. Masel, A. Marm Kilpatrick, C. Fraser, The timing of COVID-19 transmission. medRxiv 20188516 [Preprint]. 16 September 2020. 10.1101/2020.09.04.20188516.

[22] M. Marks, P. Millat-Martinez, D. Ouchi, C. Roberts, A. Alemany, M. Corbacho-Monn, M. Ubals, A. Tobias, C. Teb, E. Ballana, Q. Bassat, B. Baro, M. Vall-Mayans, C. G-Beiras, N. Prat, J. Ara, B. Clotet, O. Mitj, C. G. Beiras, N. Prat, J. Ara, B. Clotet, O. Mitj, Transmission of COVID-19 in 282 clusters in Catalonia, Spain: A cohort study. Lancet Infect. Dis. (2021). 10.1016/S1473-3099(20)30985-3 PMC7906723 33545090

[23] J. Raghwani, A. D. Redd, A. F. Longosz, C.-H. Wu, D. Serwadda, C. Martens, J. Kagaayi, N. Sewankambo, S. F. Porcella, M. K. Grabowski, T. C. Quinn, M. A. Eller, L. A. Eller, F. Wabwire-Mangen, M. L. Robb, C. Fraser, K. A. Lythgoe, Evolution of HIV-1 within untreated individuals and at the population scale in Uganda. PLOS Pathog. 14, e1007167 (2018). 10.1371/journal.ppat.1007167 30052678 PMC6082572

[24] G. Tonkin-Hill, I. Martincorena, R. Amato, A. R. J. Lawson, M. Gerstung, I. Johnston, D. K. Jackson, N. R. Park, S. V. Lensing, M. A. Quail, S. Gonalves, C. Ariani, M. S. Chapman, W. L. Hamilton, L. W. Meredith, G. Hall, A. S. Jahun, Y. Chaudhry, M. Hosmillo, M. L. Pinckert, I. Georgana, A. Yakovleva, L. G. Caller, S. L. Caddy, T. Feltwell, F. A. Khokhar, C. J. Houldcroft, M. D. Curran, S. Parmar, The COVID-19 Genomics UK (COG-UK) Consortium, A. Alderton, R. Nelson, E. Harrison, J. Sillitoe, S. D. Bentley, J. C. Barrett, M. Estee Torok, I. G. Goodfellow, C. Langford, D. Kwiatkowski, Wellcome Sanger Institute COVID-19 Surveillance Team, Patterns of within-host genetic diversity in SARS-CoV-2. bioRxiv 424229 [Preprint]. 25 December 2020. 10.1101/2020.12.23.424229.

[25] A. Popa, J.-W. Genger, M. D. Nicholson, T. Penz, D. Schmid, S. W. Aberle, B. Agerer, A. Lercher, L. Endler, H. Colao, M. Smyth, M. Schuster, M. L. Grau, F. Martnez-Jimnez, O. Pich, W. Borena, E. Pawelka, Z. Keszei, M. Senekowitsch, J. Laine, J. H. Aberle, M. Redlberger-Fritz, M. Karolyi, A. Zoufaly, S. Maritschnik, M. Borkovec, P. Hufnagl, M. Nairz, G. Weiss, M. T. Wolfinger, D. von Laer, G. Superti-Furga, N. Lopez-Bigas, E. Puchhammer-Stckl, F. Allerberger, F. Michor, C. Bock, A. Bergthaler, Genomic epidemiology of superspreading events in Austria reveals mutational dynamics and transmission properties of SARS-CoV-2. Sci. Transl. Med. 12, eabe2555 (2020). 10.1126/scitranslmed.abe2555 33229462 PMC7857414

[26] A. L. Valesano, K. E. Rumfelt, D. E. Dimcheff, C. N. Blair, W. J. Fitzsimmons, J. G. Petrie, E. T. Martin, A. S. Lauring, Temporal dynamics of SARS-CoV-2 mutation accumulation within and across infected hosts. bioRxiv 2021.01.19.427330 (2021). 10.1101/2021.01.19.427330 33826681 PMC8055005

[27] M. P. Zwart, S. F. Elena, Matters of size: Genetic bottlenecks in virus infection and their potential impact on evolution. Annu. Rev. Virol. 2, 161 179 (2015). 10.1146/annurev-virology-100114-055135 26958911

[28] A. Sobel Leonard, D. B. Weissman, B. Greenbaum, E. Ghedin, K. Koelle, Transmission bottleneck size estimation from pathogen deep-sequencing data, with an application to human influenza A virus. J. Virol. 91, e00171-17 (2017). 10.1128/JVI.00171-17 28468874 PMC5487570

[29] A. Sobel Leonard, D. B. Weissman, B. Greenbaum, E. Ghedin, K. Koelle, Correction for Sobel Leonard et al., Transmission bottleneck size estimation from pathogen deep-sequencing data, with an application to human influenza A virus. J. Virol. 93, e00936-19 (2019). 10.1128/JVI.00936-19 28468874 PMC5487570

[30] M. Ghafari, C. K. Lumby, D. B. Weissman, C. J. R. Illingworth, Inferring transmission bottleneck size from viral sequence data using a novel haplotype reconstruction method. J. Virol. 94, e00014-20 (2020). 10.1128/JVI.00014-20 32295920 PMC7307158

[31] J. T. McCrone, R. J. Woods, E. T. Martin, R. E. Malosh, A. S. Monto, A. S. Lauring, Stochastic processes constrain the within and between host evolution of influenza virus. eLife 7, e35962 (2018). 10.7554/eLife.35962 29683424 PMC5933925

[32] A. Varble, R. A. Albrecht, S. Backes, M. Crumiller, N. M. Bouvier, D. Sachs, A. Garca-Sastre, B. R. tenOever, Influenza A virus transmission bottlenecks are defined by infection route and recipient host. Cell Host Microbe 16, 691 700 (2014). 10.1016/j.chom.2014.09.020 25456074 PMC4272616

[33] Z. Shen, Y. Xiao, L. Kang, W. Ma, L. Shi, L. Zhang, Z. Zhou, J. Yang, J. Zhong, D. Yang, L. Guo, G. Zhang, H. Li, Y. Xu, M. Chen, Z. Gao, J. Wang, L. Ren, M. Li, Genomic diversity of severe acute respiratory syndromecoronavirus 2 in patients with coronavirus disease 2019. Clin. Infect. Dis. (2020). 10.1093/cid/ciaa203 PMC7108196 32129843

[34] T. H. Wang, Y. K. Donaldson, R. P. Brettle, J. E. Bell, P. Simmonds, Identification of shared populations of human immunodeficiency virus type 1 infecting microglia and tissue macrophages outside the central nervous system. J. Virol. 75, 11686 11699 (2001). 10.1128/JVI.75.23.11686-11699.2001 11689650 PMC114755

[35] K. M. Braun, G. K. Moreno, P. J. Halfmann, E. B. Hodcroft, D. A. Baker, E. C. Boehm, A. M. Weiler, A. K. Haj, M. Hatta, S. Chiba, T. Maemura, Y. Kawaoka, K. Koelle, D. H. OConnor, T. C. Friedrich, Transmission of SARS-CoV-2 in domestic cats imposes a narrow bottleneck. bioRxiv 384917 [Preprint]. 4 January 2021. 10.1101/2020.11.16.384917. PMC7946358 33635912

[36] S. E. James, S. Ngcapu, A. M. Kanzi, H. Tegally, V. Fonseca, J. Giandhari, E. Wilkinson, B. Chimukangara, S. Pillay, L. Singh, M. Fish, I. Gazy, K. Khanyile, R. Lessells, T. de Oliveira, High resolution analysis of transmission dynamics of Sars-Cov-2 in two major hospital outbreaks in South Africa leveraging intrahost diversity. medRxiv 20231993 [Preprint]. 16 November 2020. 10.1101/2020.11.15.20231993.

[37] M. A. Martin, K. Koelle, Reanalysis of deep-sequencing data from Austria points towards a small SARS-COV-2 transmission bottleneck on the order of one to three virions. bioRxiv 432096 [Preprint]. 22 February 2021. 10.1101/2021.02.22.432096.

[38] M. Gelbart, S. Harari, Y. Ben-Ari, T. Kustin, D. Wolf, M. Mandelboim, O. Mor, P. S. Pennings, A. Stern, Drivers of within-host genetic diversity in acute infections of viruses. PLOS Pathog. 16, e1009029 (2020). 10.1371/journal.ppat.1009029 33147296 PMC7668575

[39] M. D. Nowak, E. M. Sordillo, M. R. Gitman, A. E. Paniz Mondolfi, Coinfection in SARS-CoV-2 infected patients: Where are influenza virus and rhinovirus/enterovirus? J. Med. Virol. 92, 1699 1700 (2020). 10.1002/jmv.25953 32352574 PMC7267652

[40] D. Kim, J. Quinn, B. Pinsky, N. H. Shah, I. Brown, Rates of co-infection between SARS-CoV-2 and other respiratory pathogens. JAMA 323, 2085 2086 (2020). 10.1001/jama.2020.6266 32293646 PMC7160748

[41] B. Dearlove, E. Lewitus, H. Bai, Y. Li, D. B. Reeves, M. G. Joyce, P. T. Scott, M. F. Amare, S. Vasan, N. L. Michael, K. Modjarrad, M. Rolland, A SARS-CoV-2 vaccine candidate would likely match all currently circulating variants. Proc. Natl. Acad. Sci. U.S.A. 117, 23652 23662 (2020). 10.1073/pnas.2008281117 32868447 PMC7519301

[42] B. Morel, P. Barbera, L. Czech, B. Bettisworth, L. Hbner, S. Lutteropp, D. Serdari, E.-G. Kostaki, I. Mamais, A. M. Kozlov, P. Pavlidis, D. Paraskevis, A. Stamatakis, Phylogenetic analysis of SARS-CoV-2 data is difficult. Mol. Biol. Evol. msaa314 (2020). 10.1093/molbev/msaa314 33316067 PMC7798910

[43] L. van Dorp, M. Acman, D. Richard, L. P. Shaw, C. E. Ford, L. Ormond, C. J. Owen, J. Pang, C. C. S. Tan, F. A. T. Boshier, A. T. Ortiz, F. Balloux, Emergence of genomic diversity and recurrent mutations in SARS-CoV-2. Infect. Genet. Evol. 83, 104351 (2020). 10.1016/j.meegid.2020.104351 32387564 PMC7199730

[44] Q. Li, J. Wu, J. Nie, L. Zhang, H. Hao, S. Liu, C. Zhao, Q. Zhang, H. Liu, L. Nie, H. Qin, M. Wang, Q. Lu, X. Li, Q. Sun, J. Liu, L. Zhang, X. Li, W. Huang, Y. Wang, The impact of mutations in SARS-CoV-2 spike on viral infectivity and antigenicity. Cell 182, 1284 1294.e9 (2020). 10.1016/j.cell.2020.07.012 32730807 PMC7366990

[45] S. Pond. Natural selection analysis of global SARS-CoV-2/COVID-19 enabled by data from GISAID (Datamonkey, 2021); https://observablehq.com/@spond/revised-sars-cov-2-analytics-page.

[46] J. T. Herbeck, D. C. Nickle, G. H. Learn, G. S. Gottlieb, M. E. Curlin, L. Heath, J. I. Mullins, Human immunodeficiency virus type 1 env evolves toward ancestral states upon transmission to a new host. J. Virol. 80, 1637 1644 (2006). 10.1128/JVI.80.4.1637-1644.2006 16439520 PMC1367147

[47] T. N. Starr, A. J. Greaney, A. Addetia, W. W. Hannon, M. C. Choudhary, A. S. Dingens, J. Z. Li, J. D. Bloom, Prospective mapping of viral mutations that escape antibodies used to treat COVID-19. Science 371, 850 854 (2021). 10.1126/science.abf9302 33495308 PMC7963219

[48] A. J. Greaney, A. N. Loes, K. H. D. Crawford, T. N. Starr, K. D. Malone, H. Y. Chu, J. D. Bloom, Comprehensive mapping of mutations to the SARS-CoV-2 receptor-binding domain that affect recognition by polyclonal human serum antibodies. bioRxiv 425021 [Preprint]. 4 January 2021. 10.1101/2020.12.31.425021. PMC7869748 33592168

[49] K. R. McCarthy, L. J. Rennick, S. Nambulli, L. R. Robinson-McCarthy, W. G. Bain, G. Haidar, W. P. Duprex, Recurrent deletions in the SARS-CoV-2 spike glycoprotein drive antibody escape. Science eabf6950 (2021). 33536258 10.1126/science.abf6950 PMC7971772

[50] E. M. Volz, V. Hill, J. T. McCrone, A. Price, D. Jorgensen, A. OToole, J. A. Southgate, R. Johnson, B. Jackson, F. F. Nascimento, S. M. Rey, S. M. Nicholls, R. M. Colquhoun, A. da Silva Filipe, J. G. Shepherd, D. J. Pascall, R. Shah, N. Jesudason, K. Li, R. Jarrett, N. Pacchiarini, M. Bull, L. Geidelberg, I. Siveroni, I. G. Goodfellow, N. J. Loman, O. Pybus, D. L. Robertson, E. C. Thomson, A. Rambaut, T. R. Connor, Evaluating the effects of SARS-CoV-2 Spike mutation D614G on transmissibility and pathogenicity. medRxiv 20166082 [Preprint]. 1 September 2020. 10.1101/2020.07.31.20166082.

[51] E. C. Thomson, L. E. Rosen, J. G. Shepherd, R. Spreafico, A. da Silva Filipe, J. A. Wojcechowskyj, C. Davis, L. Piccoli, D. J. Pascall, J. Dillen, S. Lytras, N. Czudnochowski, R. Shah, M. Meury, N. Jesudason, A. De Marco, K. Li, J. Bassi, A. OToole, D. Pinto, R. M. Colquhoun, K. Culap, B. Jackson, F. Zatta, A. Rambaut, S. Jaconi, V. B. Sreenu, J. Nix, I. Zhang, R. F. Jarrett, W. G. Glass, M. Beltramello, K. Nomikou, M. Pizzuto, L. Tong, E. Cameroni, T. I. Croll, N. Johnson, J. Di Iulio, A. Wickenhagen, A. Ceschi, A. M. Harbison, D. Mair, P. Ferrari, K. Smollett, F. Sallusto, S. Carmichael, C. Garzoni, J. Nichols, M. Galli, J. Hughes, A. Riva, A. Ho, M. Schiuma, M. G. Semple, P. J. M. Openshaw, E. Fadda, J. K. Baillie, J. D. Chodera, S. J. Rihn, S. J. Lycett, H. W. Virgin, A. Telenti, D. Corti, D. L. Robertson, G. Snell; ISARIC4C Investigators; COVID-19 Genomics UK (COG-UK) Consortium, Circulating SARS-CoV-2 spike N439K variants maintain fitness while evading antibody-mediated immunity. Cell 184, 1171 1187.e20 (2021). 10.1016/j.cell.2021.01.037 33621484 PMC7843029

[52] M. R. Zambenedetti, D. P. Pavoni, A. C. Dallabona, A. C. Dominguez, C. O. Poersch, S. P. Fragoso, M. A. Krieger, Internal control for real-time polymerase chain reaction based on MS2 bacteriophage for RNA viruses diagnostics. Mem. Inst. Oswaldo Cruz 112, 339 347 (2017). 10.1590/0074-02760160380 28403327 PMC5398160

[53] F. Gao, D. L. Robertson, C. D. Carruthers, S. G. Morrison, B. Jian, Y. Chen, F. Barr-Sinoussi, M. Girard, A. Srinivasan, A. G. Abimiku, G. M. Shaw, P. M. Sharp, B. H. Hahn, A comprehensive panel of near-full-length clones and reference sequences for non-subtype B isolates of human immunodeficiency virus type 1. J. Virol. 72, 5680 5698 (1998). 10.1128/JVI.72.7.5680-5698.1998 9621027 PMC110237

[54] D. E. Wood, J. Lu, B. Langmead, Improved metagenomic analysis with Kraken 2. Genome Biol. 20, 257 (2019). 10.1186/s13059-019-1891-0 31779668 PMC6883579

[55] T. Golubch, Castanet (Github, 2020); https://github.com/tgolubch/castanet.

[56] A. M. Bolger, M. Lohse, B. Usadel, Trimmomatic: A flexible trimmer for Illumina sequence data. Bioinformatics 30, 2114 2120 (2014). 10.1093/bioinformatics/btu170 24695404 PMC4103590

[57] C. Wymant, F. Blanquart, T. Golubchik, A. Gall, M. Bakker, D. Bezemer, N. J. Croucher, M. Hall, M. Hillebregt, S. H. Ong, O. Ratmann, J. Albert, N. Bannert, J. Fellay, K. Fransen, A. Gourlay, M. K. Grabowski, B. Gunsenheimer-Bartmeyer, H. F. Gnthard, P. Kivel, R. Kouyos, O. Laeyendecker, K. Liitsola, L. Meyer, K. Porter, M. Ristola, A. van Sighem, B. Berkhout, M. Cornelissen, P. Kellam, P. Reiss, C. Fraser; BEEHIVE Collaboration, Easy and accurate reconstruction of whole HIV genomes from short-read sequence data with *shiver*. Virus Evol. 4, vey007 (2018). 10.1093/ve/vey007 29876136 PMC5961307

[58] Sanger Institute, Tools directory (Sanger Institute, 2021); https://www.sanger.ac.uk/science/tools.

[59] B. Langmead, S. L. Salzberg, Fast gapped-read alignment with Bowtie 2. Nat. Methods 9, 357 359 (2012). 10.1038/nmeth.1923 22388286 PMC3322381

[60] A. OToole, V. Hill, J. T. McCrone, E. Scher, A. Rambaut, Pangolin COVID-19 lineage assigner v2.1.7, lineages version 2021-01-20 (Centre for Genomic Pathogen Surveillance, 2021); https://pangolin.cog-uk.io/.

[61] C. Mavian, S. Marini, M. Prosperi, M. Salemi, A snapshot of SARS-CoV-2 genome availability up to April 2020 and its implications. JMIR Public Health Surveill. 6, e19170 (2020). 10.2196/19170 32412415 PMC7265655

[62] Y. Shu, J. McCauley, GISAID: Global initiative on sharing all influenza data - from vision to reality. Euro Surveill. 22, 30494 (2017). 10.2807/1560-7917.ES.2017.22.13.30494 28382917 PMC5388101

[63] L. Fu, B. Niu, Z. Zhu, S. Wu, W. Li, CD-HIT: Accelerated for clustering the next-generation sequencing data. Bioinformatics 28, 3150 3152 (2012). 10.1093/bioinformatics/bts565 23060610 PMC3516142

[64] K. Katoh, K. Misawa, K. Kuma, T. Miyata, MAFFT: A novel method for rapid multiple sequence alignment based on fast Fourier transform. Nucleic Acids Res. 30, 3059 3066 (2002). 10.1093/nar/gkf436 12136088 PMC135756

[65] M. Nei, T. Gojobori, Simple methods for estimating the numbers of synonymous and nonsynonymous nucleotide substitutions. Mol. Biol. Evol. 3, 418 426 (1986). 3444411 10.1093/oxfordjournals.molbev.a040410

[66] A. M. Kozlov, D. Darriba, T. Flouri, B. Morel, A. Stamatakis, RAxML-NG: A fast, scalable and user-friendly tool for maximum likelihood phylogenetic inference. Bioinformatics 35, 4453 4455 (2019). 10.1093/bioinformatics/btz305 31070718 PMC6821337

[67] X. Didelot, D. J. Wilson, ClonalFrameML: Efficient inference of recombination in whole bacterial genomes. PLOS Comput. Biol. 11, e1004041 (2015). 10.1371/journal.pcbi.1004041 25675341 PMC4326465

[68] D. T. Hoang, O. Chernomor, A. von Haeseler, B. Q. Minh, L. S. Vinh, UFBoot2: Improving the Ultrafast Bootstrap Approximation. Mol. Biol. Evol. 35, 518 522 (2018). 10.1093/molbev/msx281 29077904 PMC5850222

[69] COVID-19 Genomics UK (COG-UK) consortiumcontact@cogconsortium.uk, An integrated national scale SARS-CoV-2 genomic surveillance network. Lancet Microbe 1, e99 e100 (2020). 10.1016/S2666-5247(20)30054-9 32835336 PMC7266609

[70] Data for: K. A. Lythgoe, M. Hall, L. Ferretti, M. de Cesare, G. MacIntyre-Cockett, A. Trebes, M. Andersson, N. Otecko, E. L. Wise, N. Moore, J. Lynch, S. Kidd, N. Cortes, M. Mori, R. Williams, G. Vernet, A. Justice, A. Green, S. M. Nicholls, M. A. Ansari, L. Abeler-Drner, C. E. Moore, T. E. A. Peto, D. W. Eyre, R. Shaw, P. Simmonds, D. Buck, J. A. Todd on behalf of the Oxford Virus Sequencing Analysis Group (OVSG), T. R. Connor, S. Ashraf, A. da Silva Filipe, J. Shepherd, E. C. Thomson, The COVID-19 Genomics UK (COG-UK) Consortium, D. Bonsall, C. Fraser, T. Golubchik, SARS-CoV-2 within-host diversity and transmission. Zenodo (2021); 10.5281/zenodo.4570598.

